# Use of minimally invasive cerclage wiring for displaced major fragments of femoral shaft fractures after intramedullary nailing promotes bone union and a functional outcome

**DOI:** 10.1186/s13018-022-03439-0

**Published:** 2022-12-12

**Authors:** Xingguang Tao, Qing Yang

**Affiliations:** Department of Orthopedics, Qingpu Branch of Zhongshan Hospital Affiliated to Fudan University, Qingpu District Central Hospital Shanghai, No. 1158, East Gongyuan Road, Qingpu District, 201700 Shanghai, People’s Republic of China

**Keywords:** Femur fracture, Cerclage wire, Intramedullary nail, Minimally invasive

## Abstract

**Background:**

Femoral shaft fractures caused by high energy trauma can be very challenging due to the large variability in fracture morphology and poor functional outcomes. Displaced major fragments of femoral shaft fractures are difficult to manage after closed reduction and intramedullary nailing (IMN). The minimally invasive cerclage wiring (CW) procedure has become an optimal tool for major fragment resetting and stabilization after IMN. However, arguments continue for the potential risk of arterial injury, blood supply disruption, and delayed bone union or non-union with the CW procedure. The surgical algorithm for treating femoral shaft fractures with displaced major fragments remains controversial. Thus, emphasis is placed on whether the CW procedure can promote the bone union rate and improve functional outcomes without significant complications.

**Methods:**

We performed a retrospective study on all patients of femoral shaft fractures with displaced major fragments between June 2015 and August 2019 in our trauma centre. Eligible patients were included and stratified into the CW group and IMN group. Demographics, radiological data, callus formation, union time, and functional outcomes were critically compared between the two groups.

**Results:**

Thirty-seven patients were included in the present study according to our inclusion/exclusion criteria, of whom 16 (43.2%) were stratified into the CW group, and 21 (56.8%) into the IMN group. The modified radiographic union score for femorae (mRUSH) in the CW group and IMN group was significantly different (11.94 ± 1.29 vs. 7.95 ± 0.74, 6 months; 15.88 ± 0.50 vs. 10.33 ± 0.91, 12 months) (*p* < 0.0001). The mean union time was significantly different between the CW and IMN groups (7.9 ± 3.2 months vs. 20.1 ± 8.48 months) (*p* < 0.0001). Bone union at 12 months differed significantly between the CW and IMN groups (15 vs. 5) (*p* < 0.05). The Harris Hip Score in the CW group was significantly higher than that in the IMN group (88.19 ± 4.69 vs. 76.81 ± 5.26, 12 months; 93.19 ± 4.68 vs. 87.57 ± 5.38, 24 months) (*p* < 0.01). The Hospital for Special Surgery Knee Score was significantly different between the CW and IMN groups (78.50 ± 5.65 vs. 67.71 ± 4.65, 12 months; 89.50 ± 5.05 vs. 75.81 ± 8.90, 24 months) (*p* < 0.0001).

**Conclusions:**

Minimally invasive CW is an optimal supplement for IMN in the treatment of femoral shaft fractures with displaced major fragments. As illustrated, the benefits of CW potentially include promotion of the bone union rate and improvement in functional outcomes.

## Introduction

Femoral shaft fractures featuring various fracture morphologies and displaced major fragments (10–34%) [1, 2] still remain a challenge for trauma surgeons. For all femoral shaft fractures the main goal of treatment is to restore cortical congruence, and alignment, and achieve sufficient fracture stability that allows early mobilization. Although intramedullary nailing (IMN) has been widely used to restore fracture alignment through closed reduction, displaced major fragments are difficult to manage in many cases. Subsequently, prolonged bone union time [3, 4], poor functional outcomes [5], and even nail breakage may occur. Therefore, reduction of the displaced major fragments and reinforcement of stabilization are crucial. Thus, minimally invasive cerclage wiring (CW) has introduced to minimize the gaps of the fragments and reinforce the stability of the nails in the treatment of comminuted femoral shaft fractures [6–8].

Previous studies have reported that stabilization with IMN and CW had no effect on the bone union rate of femoral fractures [6, 9] and offered excellent outcomes [6, 8]. However, only a few performed thorough validation to evaluate the outcome of CW versus IMN in the treatment of femoral shaft fractures.

The goal of this retrospective study was to compare the outcomes of CW and/or IMN for femoral shaft fractures with displaced major fragments. We also discuss the facility of use of CW for trauma surgeons and determine the value of CW regarding not only the bone union rate but also functional outcomes.

## Materials and methods

### Patients

This retrospective comparative study, conducted from June 2015 to August 2019 in our level 1 trauma centre, was approved by the institutional review board (approval no. 2020-47). The inclusion criteria were as follows: (1) age ≥ 18 years, (2) comminuted femoral shaft fractures with displaced major fragments (including fracture lines extended to the subtrochanteric zone and metaphysis of the distal femur), and (3) previous minimally invasive CW and/or IMN procedures. The exclusion criteria were as follows: (1) age > 65 years, (2) open fractures, (3) femoral shaft fractures without major displaced fragments, (4) chronic diseases affecting bone union, and (5) inadequate medical records. Finally, a total of 37 patients were included in this study and demographics. (Age, sex, radiological data, AO fracture type, surgical methods, bone union time, postoperative complications, and functional outcomes were retrospectively collected from our trauma centre database (Table 1)).

**Table 1:** Demographic data of patients in the current study

Demographic data	Subjects (*n* = 37)
Age	44.2 ± 15.9
*Sex*	
Male	23
Female	12
*Side*	
Right	23
Left	14
*Location*	
Proximal	18
Middle	14
Distal	5
*L* (cm)	9.2 ± 4.4
Dprox (cm)	2.5 ± 1.1
Ddist (cm)	3.3 ± 2.3
Bd (cm)	2.8 ± 0.3
*AO/OTA*	
32B	38
32C	9
IMN	21
Anterograde	19
Retrograde	2
IMN + CW	16
Anterograde	15
Retrograde	1
1CW	11
2CW	5
Follow-up duration	27.78 ± 3.00

*L* length of the major fragment, *Dprox* proximal displacement of the major fragment, *Ddist* distal displacement of the major fragment, *Bd* diameter of femur, *IMN* intramedullary nailing, *CW* cerclage wiring

### Data collection and study protocol

Patients were stratified into the IMN group (intramedullary nails, *n* = 21) or CW group (intramedullary nails and cerclage wires, *n* = 16) according to the method of fracture fixation. The surgical planning for femoral shaft fractures is described below.

**Fig. 1:**
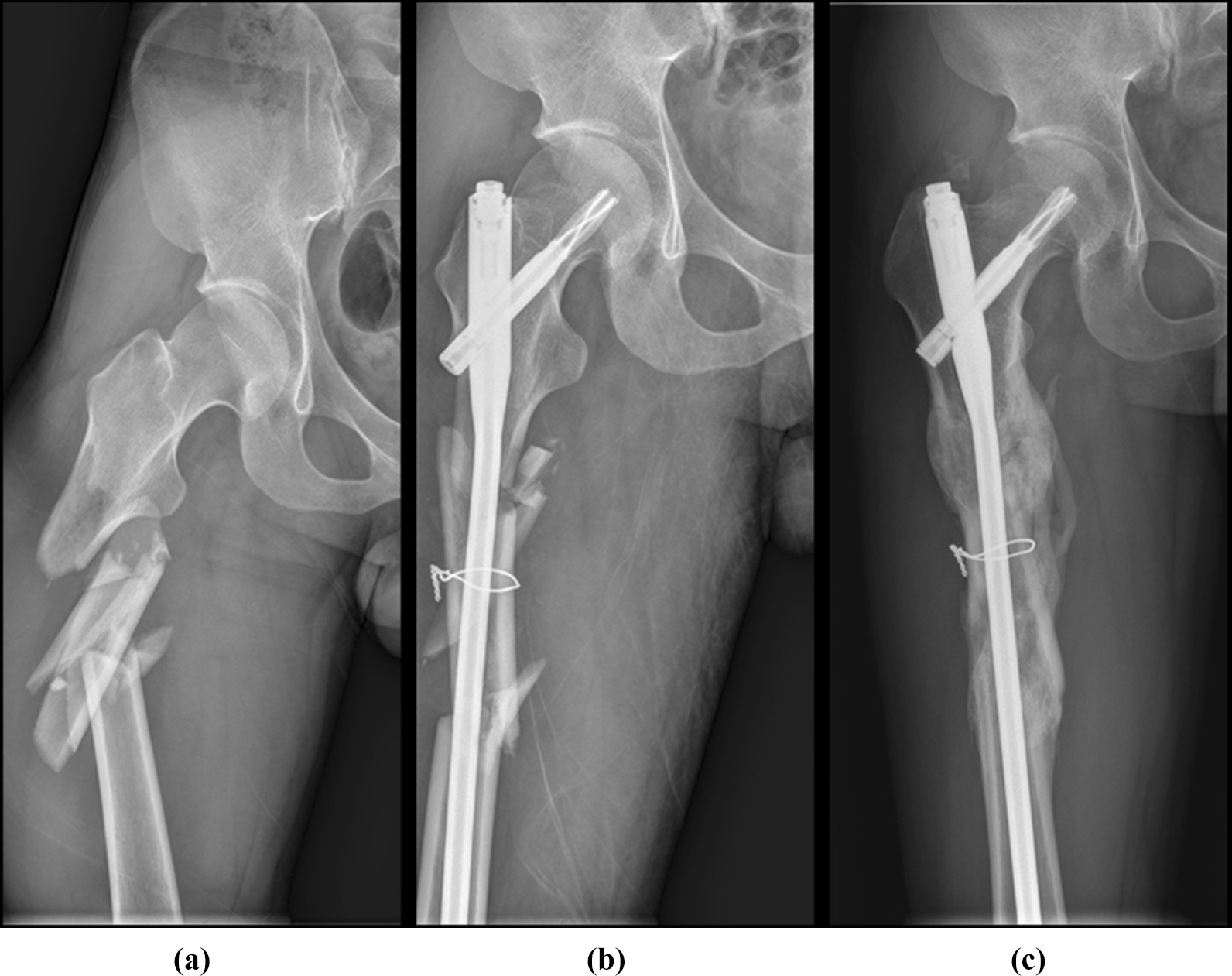
**a** A comminuted femoral shaft fracture with a displaced major fragment. **b** After 11 days, a cerclage wire was used after intramedullary nailing to reset and stabilize the major fragment of femoral shaft fracture. **c** Radiological image showed bone union after a period of 8-month follow-up

**Fig. 2:**
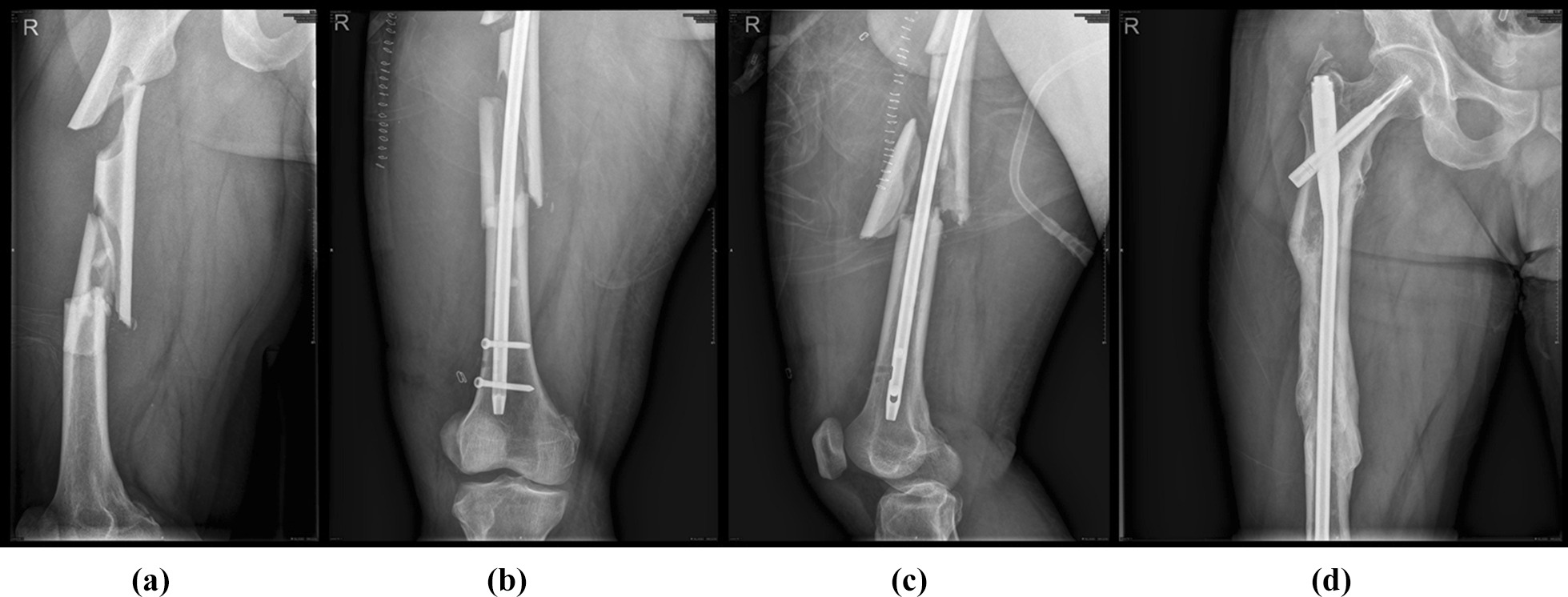
**a** A comminuted femoral shaft fracture with displaced major fragments. **b** After 13 days, intramedullary nailing was performed to stabilize the comminuted femoral shaft fracture with residual displaced major fragments on the anterior–posterior image. **c** Displacement of the major fragments was more pronounced (> 1 cm) on the lateral image. **d** After a period of 24-month follow-up, the X-ray image showed bone union of the femoral shaft fracture with displaced major fragments

All patients were placed on a universal orthopaedic traction bed in a supine position. Intramedullary nails were used in an antegrade or retrograde manner according to fracture locations in the IMN group (Fig. 1), while one or two cerclage wires were minimally utilized through a cannulated semicircular wire passer [10] to reset and stabilize the displaced major fragments after closed reduction by IMN and other surgical techniques in the CW group (Fig. 2). Postoperative rehabilitation was encouraged for all individuals, and the use of weight-bearing was evaluated and permitted by trauma surgeons. All patients were followed up until bone union, which was evaluated by X-ray examinations.

The length of the major fragment (L), proximal or distal displacement (Dprox, Ddist), and diameter of the femur (Bd) were reviewed and recorded according to X-ray radiographs. Dprox and Ddist were defined as the displacement from the ends of a displaced major fragment to the centre of the femoral cavity on X-ray images.

Callus formation was assessed using the modified radiographic union score for femorae (mRUSH). Postoperative complications including neurovascular injury, infection, delayed union or non-union, and internal fixation failure were reviewed. Union time was defined as the disappearance of the fracture line, cortical continuity, and the surgeon’s general impression [11]. Delayed union was defined as union after more than 10 months if bone callus was not present in more than three of the four cortical bone surfaces via the frontal and lateral radiographic views.

Functional outcomes of the hip and knee were evaluated by the Harris hip score (HHS) (≥ 90 excellent; 80–89 good; 70–79 fair; < 70 poor) and Hospital for Special Surgery Knee Score (HSS) (excellent ≥ 80, good 70–79, fair 60–69, poor < 59), respectively. Demographics (age, sex, fracture type, fracture location, and radiological data) were reviewed and compared to investigate whether there was significant difference between the two groups. Postoperative factors (complications, mRUSH, union time, HHS, and HSS) were analysed by group comparison to evaluate the value of CW as a supplement to IMN.

### Statistical analysis

The Pearson chi-square test and the t test were used to investigate the grouping variables. Statistical analysis was performed using SAS software (version 9.1; SAS Institute, INC., Cary, NC). *p* < 0.05 was considered to indicate a statistically significant difference.

## Results

The mean age of the patients was 44.2 ± 15.9 years, and there were 23 males and 12 females. Twenty-three patients had fractures of the right femoral shaft, while 19 had fractures of the left. Femoral fractures were located in the proximal (18), middle (14), and distal (5) regions. Thirty-eight fractures were categorized as AO B type, and 9 were categorized as C type. In the IMN group, there were 19 anterograde nails and 2 retrograde nails, while in the CW group, there were 15 anterograde and 6 retrograde nails. One cerclage wire was used in 11 patients, and two cerclage wires were utilized in 5.

The mean length of the major fragments was 9.2 ± 4.4 cm, while the mean Dprox and Ddist were 2.5 ± 1.1 cm and 3.3 ± 2.3 cm, respectively. Demographics (age, sex, fracture type, fracture location, and radiological data) were not significantly different between the two groups (*p* > 0.05) (Table 2).

**Table 2:** Comparison demographics of cerclage wiring group and intramedullary nailing group

	IMN group (*n* = 21)	CW group (*n* = 16)	*p* value
Mean age (S.D.)	42.4 ± 16.5	45.6 ± 15.7	0.5540 > 0.05
*Sex (n, %)*			
Male	15	10	0.5654 > 0.05
Female	6	6	0.5654 > 0.05
*AO/OTA pattern (n)*			
32B	16	12	0.9334 > 0.05
32C	5	4	0.9334 > 0.05
*L* (cm)	8.7 ± 5.3	9.7 ± 3.0	0.4915 > 0.05
Dprox (cm)	2.6 ± 1.2	2.3 ± 0.9	0.5610 > 0.05
Ddist (cm)	2.8 ± 1.6	3.9 ± 2.9	0.1468 > 0.05
Bd	2.8 ± 0.3	2.7 ± 0.3	0.8547 > 0.05
*Site (n)*			
Middle	9	5	0.4708 > 0.05
Distal or proximal	12	11	0.4708 > 0.05

*L* length of the major fragment, *Dprox* proximal displacement of the major fragment, *Ddist* distal displacement of the major fragment, *Bd* diameter of the femur, *IMN* intramedullary nailing, *CW* cerclage wiring

*p < 0.05

After a period of 27.78 ± 3.00 months of follow-up, complications (neurovascular injuries, infection, and internal fixation failure) were not observed in the present study. mRUSH was significantly different between the two groups (7.95 ± 0.74 vs. 11.94 ± 1.29 at 6 months, *p *< 0.0001; 10.33 ± 0.91 vs. 15.88 ± 0.50 at 12 months, *p* < 0.0001). The mean union time was analysed by group comparison and was significant (20.1 ± 8.48 months vs. 7.9 ± 3.2 months). Bone union was achieved in the CW and IMN groups (5 vs. 1, 12 months, *p* = 0.0215; 16 vs. 16, 24 months, *p* = 0.0358). No one was observed to suffer delayed union or non-union in the two groups. No reoperation was performed in the current study (Table 3).

**Table 3:** Comparison of the union time between intramedullary nailing group and cerclage wiring group

	IMN group (*n* = 21)	CW group (*n* = 16)	*p* value
^a^Score at 6 months*	7.95 ± 0.74	11.94 ± 1.29	< 0.0001
^a^Score at 12 months*	10.33 ± 0.91	15.88 ± 0.50	< 0.0001
Mean union time (months)*	20.1 ± 8.48	7.9 ± 3.2	< 0.0001
Union in 12 months (*n*)*	5	15	0.0216
Union in 24 months (*n*)*	16	16	0.0358
Delayed union (*n*)	0	0	
Reoperation (*n*)	0	0	

Data was expressed as mean (S.D.)

*IMN* intramedullary nailing, *CW* cerclage wiring

^a^Modified radiographic union score for femorae (mRUSH)

*p < 0.05

The HHS in the CW group was 88.19 ± 4.69, which was higher than that in the IMN group (76.81 ± 5.26) at 12 months (*p* < 0.0001). This was also the case at 24 months (93.19 ± 4.68 vs. 87.57 ± 5.38) (*p *< 0.01). The HSS in the CW and IMN groups was significantly different (78.50 ± 5.65 vs. 67.71 ± 4.65, 12 months; 89.50 ± 5.05 vs. 75.81 ± 8.90, 24 months) (*p* < 0.0001) (Table 4). HHS rated excellent (0 vs. 4, *p* = 0.0153, 12 months; 5 vs. 13, *p* < 0.0001, 24 months), good (5 vs. 12, *p* = 0.0020; 16 vs. 3, *p* = 0.0005) and HSS rated excellent (0 vs. 5, *p* = 0.0172, 12 months; 4 vs. 15, *p* < 0.0001, 24 months), good (5 vs. 11, *p* = 0.0063, 12 months; 11 vs. 1, *p* = 0.0003, 24 months). The number of excellent and good HHS score was significant (5 vs. 16, *p* < 0.0001, 12 months) as was the number of excellent and good HSS scores (5 vs. 16, *p* < 0.0001; 15 vs. 16, *p* = 0.0195) (Table 5).

**Table 4:** Scores comparison of HHS and HSS in the CW and IMN groups

	IMN group (*n* = 21)	CW group (*n* = 16)	*p* value
*12 months (n)*			
HHS	76.81 ± 5.26	88.19 ± 4.69	< 0.0001
HSS	67.71 ± 4.65	78.50 ± 5.65	< 0.0001
*24 months (n)*			
HHS	87.57 ± 5.38	93.19 ± 4.68	0.0018
HSS	75.81 ± 8.90	89.50 ± 5.05	< 0.0001

*IMN* intramedullary nailing, *CW* cerclage wiring, *HHS* Harris hip score, *HSS* Hospital for Special Surgery Knee Score

**Table 5:** Comparison of functional outcomes between subjects in CW and IMN groups

	IMN group (*n* = 21)	CW group (*n* = 16)	*p* value	*p* value
*12 months (n)*				
HHS				
Excellent*	0	4	0.0153	< 0.0001
Good*	5	12	0.0020	
Fair	16	0		
Poor	0	0		
HSS				
Excellent*	0	5	0.0172	< 0.0001
Good*	5	11	0.0063	
Fair	16	0		
Poor	0	0		
*24 months (n)*				
HHS				
Excellent*	5	13	< 0.0001	–
Good*	16	3	0.0005	
Fair	0	0		
Poor	0	0		
HSS				
Excellent*	4	15	< 0.0001	0.0195
Good*	11	1	0.003	
Fair	6	0		
Poor	0	0		

*IMN* intramedullary nailing, *CW* cerclage wiring, Hospital for Special Surgery Knee Score (HSS): excellent ≥ 80, good 70–79, fair 60–69, poor < 59; Harris hip score (HHS): ≥ 90 excellent; 80–89 good; 70–79 fair; < 70 poor

*p < 0.05

## Discussion

Our results demonstrated that minimally invasive CW is a useful tool and that patients with comminuted femoral shaft fractures would benefit from both the promotion of union and improvement in functional outcome with this procedure.

CW has been used for many years as a method of reinforcing fixation but is associated with a high rate of complications including reoperations (6.6–26.1%), non-unions (3–22.0%), deep infections (3.4–13.0%), knee stiffness (4.3–10%), and refractures (9.0%) [12, 13].

CW, however, as emerged as a more minimally invasive procedure due to the advantages of the newly designed wire pass system [10] and it seems to be easier to gain optimal outcomes. However, arguments continue for potential arterial injury and the disruption of periosteal blood supply. In addition, the extent to which CW may promote the bone union rate and improve functional outcomes has not been well addressed.

Regarding the duration of bone union, CW might not affect that of femoral shaft fractures [6, 9]. In the present study, the demographics of the two groups were analysed and intergroup bias was eliminated. The presence of displaced major fragments of femoral shaft fractures after IMN could delay the course of bone union. Studies have revealed that a fragment > 8 cm [14], a fragmentary displacement of > 1 cm [15], or of > 2 cm in the proximal area [14], or > 1 cm in the distal area [14] in femoral shaft fractures after IMN affects the bone union rate. In this current study, the mean length of the fragments was 9.7 cm and 8.7 cm in the CW group and IMN group, respectively. The Dprox values of the CW and IMN groups were 2.6 cm and 2.3 cm (> 2 cm) [14], while the Ddist values were 3.9 cm and 2.8 cm (> 1 cm), respectively [15]. In our study, radiological review of femoral shaft fractures showed no significant difference between the two groups. Both the length of the fragments and fragmentary displacements in this study indicated a high risk of delayed union or non-union and the need for fragment resetting. The CW group had a shorter union time than the IMN group (7.9 months vs. 20.1 months, *p* < 0.0001). Our results were similar to previous reports [14, 15]. CW is of great value in the resetting and stabilization of displaced major fragments after IMN. Our results thus proved the point mentioned above.

Evidence can be observed by the stimulative callus formation from radiological images in the CW group, which was evaluated by mRUSH in this study. The IMN group and CW group scored 7.95 versus 11.94 at 6 months, and 10.33 versus 15.88 at 12 months, respectively (*p* < 0.0001). These radiological results were determined to be significant. This is similar to one study that reported that the mRUSH was 5.8 versus 7.6 at 6 months, and 8.6 versus 10.6 at 12 months between the large gap group (fragment displacement > 1 cm) and the small gap group (< 1 cm) [15]. Theoretically, greater callus formation in the CW group is derived from excellent reduction of major fragments, minimal disruption of blood supply, and improved microenvironment [16].

Disruption of the blood supply by the femoral shaft fracture itself has been studied. Two per cent of patients with femoral shaft fractures suffered arterial injury [17], often at the junction of the middle and distal thirds of the femoral shaft [18], and might result in ischaemia of the thigh [19]. This has inspired trauma surgeons to notice the placement of cerclage passers according to the procedure, remain vigilant for intraoperative arterial injury, and repair it immediately [20, 21].

In contrast, a cadaveric injection study showed that percutaneous CW resulted in minimal disruption of the femoral blood supply and partial disruption could be compensated [10]. After further investigation of the CW procedure [22] and technical progression for use of this technique, danger zones began to be addressed with care and more minimally invasive techniques [23]. Another study revealed that percutaneous cerclage cable fixation can provide both anatomical reduction and increased stability, while preserving the biology around the fracture site in the treatment of complex subtrochanteric fractures [24]. In the current study, the results showed no arterial injury or wire-related complications, which demonstrated that the minimally invasive technique was safe in the clinical treatment of femoral shaft fractures.

To our knowledge, few studies have scored the functional outcome of the hip or knee in the treatment of femoral shaft fractures. It was reported that as many as 15% of patients who had comminuted femoral shaft fractures could not gain satisfactory flexion of the knee by the use of IMN and CW [6]. Another study found that more than 10% of patients had no good range of motion for the hip or knee [13].

In this study, the HHS and HSS were introduced to evaluate the functional outcomes of the hip and knee. The results of this study showed that the CW group had a higher HHS and HSS than the IMN group at 12 and 24 months. Our results also showed that the excellent and good rate of HSS in the CW group (100%) was higher than that in the IMN group (71.4%) at 24 months, while the corresponding rate for the HHS was the same. Although CW is a surgical procedure, this technique has been proven to be beneficial in improving functional outcomes of the limbs.

However, the present study had certain limitations. First, a small number of patients were included in our cohort, and further analysis was infeasible. Second, we expanded the academic definition [25] of the femoral shaft to the subtrochanteric zone and metaphysis of the distal femur; however, our study focused on optimal surgical therapy clinically, and thus our definition may not be sufficiently rigorous in some aspects [26, 27]. In addition, the results of this study regard the validity of the procedure; however, longer-term investigations and larger patient groups are required to improve the confidence in the data.

## Conclusions

Minimally invasive CW is an optimal supplement for IMN in the treatment of femoral shaft fractures with displaced major fragments. As illustrated herein, severely injured patients would benefit from the CW procedure in terms of promotion of the bone union rate and improvement in the functional outcomes of the limb.

## Data Availability

All data are available without restriction contacting the corresponding authors.

## Abbreviations

IMN: Intramedullary nailing
CW: Cerclage wiring
mRUSH: Modified radiographic union score for femorae
HHS: Harris hip score
HSS: Hospital for special surgery knee score
L: Length of the major fragment
Dprox: Proximal displacement of the major fragment
Ddist: Distal displacement of the major fragment
Bd: Diameter of the femur
